# The American Year-Book of Medicine and Surgery for 1901

**Published:** 1901-05

**Authors:** 


THE AMERICAN YEAR-BOOK OF MEDICINE AND SURGERY FOR 1901. A yearly digest of scientific progress and authoritative opinion in all branches of medicine and surgery, drawn from journals, monographs and text-books of the leading American and foreign authors and investigators. Arranged with critical editorial comments, by eminent American specialists. In two volumes—Volume I, including *General Medicine,* octavo, 681 pages, illustrated; Volume II, *General Surgery,* octavo, 610 pages, illustrated. Philadelphia and London: W. B. Saunders & Co., 1901. Per volume: Cloth, $3.00 net; Half Morocco, $3.75 net.

Saunder's American Year-Book is already deservedly popular with the profession, and it is sure to continue to be so as long as it is kept up to its present high standard of excellence. This year's issue is published in two volumes, one on general medicine and the other on surgery. The various sections of these volumes are under the editorial management of specialists, most of whom are already known to the profession. The critical comments by these editors add greatly to the value of the work, and make it one of the best of its kind, and one especially well adapted to the use of the general practitioner, who for obvious reasons is unable to select for himself all that is worth reading from the great number of monographs, journals and text-books of the year.

